# Microbial Control of Invasive Forest Pests with Entomopathogenic Fungi: A Review of the Current Situation

**DOI:** 10.3390/insects10100341

**Published:** 2019-10-12

**Authors:** Surendra K. Dara, Cristian Montalva, Marek Barta

**Affiliations:** 1 University of California Cooperative Extension, University of California, 2156 Sierra Way, Ste. C, San Luis Obispo, CA 93401, USA; 2 Laboratorio de Salud de Bosques, Instituto de Conservación, Biodiversidad y Territorio, Facultad de Ciencias Forestales y Recursos Naturales, Universidad Austral de Chile, Valdivia 5090000, Chile; cristian.montalva@uach.cl; 3 Institute of Forest Ecology, Slovak Academy of Sciences, Akademická 2, 949 01 Nitra, Slovakia; marek.barta@savba.sk

**Keywords:** microbial control, entomopathogenic fungi, invasive pests, forest insects

## Abstract

The health of the forestlands of the world is impacted by a number of insect pests and some of them cause significant damage with serious economic and environmental implications. Whether it is damage of the North American cypress aphid in South America and Africa, or the destruction of maple trees in North America by the Asian long horned beetle, invasive forest pests are a major problem in many parts of the world. Several studies explored microbial control opportunities of invasive forest pests with entomopathogenic bacteria, fungi, and viruses, and some are successfully utilized as a part of integrated forest pest management programs around the world. This manuscript discusses some invasive pests and the status of their microbial control around the world with entomopathogenic fungi.

## 1. Introduction

Globalization of trade and travel directly or indirectly contributed to the spread of several insects to new areas where they have become serious pests. Invasive pests of forests not only cause economic damage, but also have an impact on the ecosystem, regionally or nationally. Use of chemical pesticides has been the primary pest control strategy for the past several decades. Due to the environmental and human health risks from excessive use of chemical pesticides, there are renewed appeals for effective, safe, and economically acceptable alternatives. Integrated pest management (IPM) emerged as an approach to address the safety issue by taking all pest management options into consideration and promoting a balanced strategy that is environmentally sustainable, economically viable, and socially acceptable and applicable various scenarios from crop pests to forest pests [1]. There are several invasive Coleoptera, Hemiptera, Hymenoptera, and Lepidoptera in forest ecosystems that have been a target of various management practices including microbial control with native, or introduced, entomopathogens. Entomopathogenic fungi are a group of phylogenetically diverse heterotrophic and eukaryotic microorganisms that are pathogens of insects and use them as hosts to develop a part of their life cycle [2,3]. Today, there are over 700 recognized species of entomopathogenic fungi representing the kingdoms of Chromista and Fungi [4]. However, a majority of important species belongs to the phylum Ascomycota (order: Hypocreales) and Entomophthoromycota (orders: Entomophthorales and Neozygitales). In general, these fungi are considered excellent candidates for microbial control of various insect pests [5,6,7]. Nonetheless, only a small number of taxa, most notably *Beauveria bassiana* (Bals. -Criv.) Vuill., *B. brongniartii* (Sacc.) Petch, *Metarhizium anisopliae* (Metschn.) Sorokin, *Lecanicillium lecanii* (Zimm.) Zare and W. Gams and *Isaria fumosorosea* Wize are in active production, sale, and general use as microbial control agents mainly in crop production systems [8]. While earlier reviews explained the importance of entomopathogens in controlling forest pests [9], the current review focused on various entomopathogenic fungi against invasive forest pests around the world.

## 2. Invasive Forest Insect Pests

The number of invasions by non-indigenous forest pests is increasing worldwide due to growing travel and trade [10]. Pest invasions consist of three phases: arrival at a site, establishment at that location, and subsequent spread [11]. Given the species richness and the wide involvement in ecosystem processes of insects, it is not surprising that they are also prominent as invasive species, both in terms of their number and their impact [12,13]. Some important examples of invasive forest insect pests, their damage, spread and the current status of microbial control are discussed here.

### 2.1. Cypress Aphid

*Cinara cupressi* Buckton, (Hemiptera: Aphididae), native of North America, belonging to a complex of several anatomically similar species [14], is currently widespread all over the world. It is exclusively associated with conifers in the Cupressaceae and Pinaceae families. These aphids feed on smaller twigs in the foliated parts of the crown and frequently cause branch die-back, resulting in damage to natural forests and plantations [15,16,17]. The aphid inserts its buccal stylet into the tree until it reaches the phloem and ingests large quantities of phloem sap, which is rich in sugars. A secondary problem caused by aphid feeding is the secretion of copious quantities of honeydew that promotes the growth of sooty mold [18,19,20,21]. Aphid feeding decreases photosynthesis and increases respiration, resulting in chlorosis of foliage and stunted growth, especially in young trees [18,22]. Several studies have pointed out the high economic and environmental impacts of cypress aphid infestations. In Kenya, 12% of the trees were killed over two years, causing significant economic losses [23]. In the southern and eastern African regions, *C. cupressi* caused a loss of $27.5 million in 1991 with a continued annual loss of $9.1 million [24]. Additionally, *C. cupressi* is also a threat to an endangered *Widdringtonia* species in Africa [25]. *Cinara cupressi* damage has also been reported in South America in the Chilean cedar, *Austrocedrus chilensis* (D. Don) Pic. Serm. and Bizzarri, and the Patagonian cypress, *Fitzroya cupressoides* I. M. Johnst. [17,25,26]. To minimize the impact of *C. cupressi* on *A. chilensis*, several governmental agencies promote a pest management program with an emphasis on biological control [17,25].

### 2.2. Eucalyptus Weevil

*Gonipterus platensis* Marelli (Coleoptera: Curculionidae), a native of Australia, has been accidentally introduced in other parts of the world where it became a serious pest of eucalyptus [27,28,29]. Based on morphological and molecular data, *G. platensis* is now recognized as a part of a cryptic species complex known as *Gonipterus scutellatus* Gyllenhal [28]. *Gonipterus scutellatus* species complex invaded countries, including France, Portugal, Italy, and Spain, and is categorized as a quarantine pest listed in Annex IIB of Council Directive 2000/29/EC [27]. As the major eucalyptus pest, it causes significant damage to eucalyptus trees around the world. The larvae feed on young leaves and defoliate the top parts of the canopy [28], while adults feed on the edges of mature leaves, impairing the growth of the tree [29]. The damaged trees show symptomatic scalloped leaf edges, with a resultant die-back of shoot tips and the development of epicormic shoots [27,30]. Damage initially appears as a brownish scorched appearance of young foliage and eventually leads to the destruction of young twigs and buds. Severe defoliation gives the trees a stunted and stag-headed appearance. Eucalyptus plantations are the most productive forest stands in Spain with around 500,000 ha of cultivated area where *Eucalyptus globulus* Labill. is the dominant species in North and North-west Spain [31]. Since 1991, the high productivity of this eucalyptus species has been threatened by outbreaks of *G. scutellatus*. It has been estimated that tree growth is sometimes reduced by 30% in Galicia [31]. Determination of the impact of different levels of defoliation on wood production is difficult because it depends on tree age, tree health status, soil parameters, and orientation of the stands [32]. Mature and healthy trees could be more tolerant to defoliation: by using an empirical growth model, it has been predicted that for 10-year-old trees the 75% and 100% defoliation would produce wood volume losses of 43% and 86%, respectively [32]. However, 20% defoliation of 3-year-old *E. globulus* results in significant reduction of stem growth within just one year after defoliation [33].

### 2.3. Gypsy Moth

Gypsy moth, *Lymantria dispar* L. (Lepidoptera: Erebidae), is native to Eurasia and North Africa and is one of the most important pests of deciduous trees in Europe spreading from west to east and from north to south [34,35]. Regular outbreaks are very common, especially in the Balkan Peninsula. Since *L. dispar* was accidentally introduced from France to the United States near Boston, its distribution has continued to expand due to favorable environmental conditions in the pest’s new home [35,36]. The extremely broad host range for larval feeding and the non-discriminating oviposition behavior of females has allowed *L. dispar* to disperse and become established through much of northeastern United States. At present, it is considered one of the most destructive forest insects in the eastern United States [35,37]. *Lymantria dispar* is also a global threat to both commercial and native forest systems due to its host range [38,39,40,41]. In the United States, the economic impacts of one subspecies, the European gypsy moth (*L. dispar dispar* L.), is estimated to be in excess of $250 million per year [42], and this is likely to increase as this species continues to spread through North America. Two other subspecies, the Asian gypsy moth (*L. dispar asiatica* Vinkovskij), found in China, the Korean peninsula and far East Russia, and the Japanese gypsy moth (*L. dispar japonica* Motschulsky), found in Japan, have not yet established outside their native range, but are of significant global concern [41]. The ecological implications of *L. dispar* defoliation include changes in forest succession patterns and watershed characteristics, stand patchiness, and sporadic masting, all of which can affect wildlife distribution patterns [43,44]. In urban landscapes, human health concerns are associated with high populations of mobile caterpillars with urticating hairs, as well as copious frass production [38].

### 2.4. Asian Longhorned Beetle

*Anoplophora glabripennis* Motschulsky (Coleoptera: Cerambycidae) is a destructive polyphagous woodborer that attacks and kills healthy trees native to Asia [45,46]. It has become a serious forest pest in China since the 1980s, as a result of the planting of vast forest and urban monocultures dominated by non-native *Populus* and *Salix* [47,48]. Between 1980 and 1990, widespread outbreaks of *A. glabripennis* occurring in Ningxia Province and Inner Mongolia led to the destruction of over 90 million infested trees [49]. To date, this insect continues to be particularly problematic in landscapes such as agricultural windbreaks, roadside greenways, plantations, and urban street trees [50]. In the last two decades, as international trade increased between China and western countries, numerous accidental introductions of *A. glabripennis* occurred in North America and Europe. It was first intercepted in the United States and Canada in 1992, on wood packaging material, and an established population was found in North America in 1996. Maple trees (*Acer* spp.) are the most commonly infested by *A. glabripennis* in both these countries. In Europe, the first *A. glabripennis* infestation was found in north-west Austria in 2001 [51]. Since then, it has been detected in France, Germany, Finland, Montenegro, Switzerland, the Netherlands and the United Kingdom [52,53,54,55,56]. Adults feed primarily on the bark and phloem of 2–3-year-old twigs and leaf petiole. Females bore into the cambial region to deposit eggs individually [57]. Larvae feed solitarily beneath the bark along the phloem–cambium interface in the early instars before boring into and feeding on the heartwood. Larvae move upward through sapwood and heartwood, forming galleries as they develop. Adults chew through the bark and exit the galleries. Due to its economic and long-term ecological damage, *A. glabripennis* is considered a serious pest.

In Italy, the citrus longhorned beetle, *Anoplophora chinensis* Forster, a related polyphagous species, was detected in the Milan area in 2000 [58]. Many ornamental trees in Lombardy region were severely affected by this pest, which was introduced through bonsai plant imported from the Far East. Major monitoring and eradication efforts began in 2004 [59,60]. Until now, the species has been observed in Italy, France, Croatia, Germany and Switzerland [59,61,62]. Although intercepted in the United States a few times, this pest has not yet been established in the United States or Canada [63].

### 2.5. Emerald Ash Borer

*Agrilus planipennis* Fairmaire (Coleoptera: Buprestidae) is an invasive tree-boring beetle native to temperate northeastern Asia, including China, Korea, and Russia. It was accidentally introduced in North America in 2002 and has currently spread to a range that now includes 31 states and 3 provinces [64,65]. It killed millions of ash trees in the United States and Canada, causing extensive ecological and economic damage [66]. In Europe, it was first recorded in 2003 in Moscow, Russia and spread 460 km south and 250 km west in the past decade [67,68]. All ash species native to Europe and North America are susceptible to *A. planipennis*, although to varying levels. While the black, green, and white ash species are the most susceptible, the white ash is less preferred, and the blush ash is the most resistant in North America. *Agrilus planipennis* causes progressive canopy decline [69]. Adults feed on foliage and females deposit eggs under the bark or within the cracks in the bark. Larvae cause rapid tree mortality via their feeding in the cambial and phloem tissue, which creates serpentine galleries that sever sap transport between shoots and roots, disrupting the water and nutrient supply [70,71]. Infested trees usually die within 2–6 years [72]. Economic consequences associated with this pest are also significant. Following spread of *A. planipennis* in Canada, the potential cost of treating ca 1.2 million ash trees in urban landscapes was estimated to be $890 million in 2010 [73]. Cost prediction for treatment following similar spread of *A. planipennis* in urban centers of 25 states in the eastern United States is $10.7 billion [74]. In addition to the economic losses to forests, properties, and affiliated industries by the mortality of trees, the pest also caused significant ecological losses by disrupting the species composition, nutrient cycles, and contributing to the spread of unwanted invasive species [42,75].

### 2.6. Oak Lace Bug

The Nearctic species *Corythucha arcuata* (Say) (Hemiptera: Tingidae), native to North America, is among one of the most important pests of oak trees (*Quercus* spp.) in forest, urban, and rural areas worldwide [76,77,78,79]. *Corythucha arcuate* was first detected in North Italy in 2000 [80] and continued to spread in Europe with a current distribution in south to central Europe, Turkey, Russia, and Iran [77,81,82,83,84]. Adults and nymphs feed on the lower side of the leaves of host trees producing numerous characteristic black spots. Corresponding upper leaf surfaces develop discoloration and whitish blotching or stippling. Due to the leaf damage, photosynthesis and respiration are reduced. Under heavy infestations, premature leaf fall may occur [77,85,86,87]. In Turkey, a few years after the first record, the oak lace bug affected an area of about 28,116 km^2^ [87]. In Bulgaria, just five years after the first recorded *C. arcuata* invaded most of the country, about 85% of the leaves displayed discoloration [88]. Besides oaks, *C. arcuata* can also occasionally attack hosts from the genera *Acer*, *Castanea*, *Malus*, *Pyrus* and *Rosa* [89,90]. In 2001, *C. arctuata* was added to the alert list of the European and Mediterranean Plant Protection Organization and subsequently deleted in 2007. The main reason for deletion was that no efficient phytosanitary measures could stop the natural spread of this species.

## 3. Microbial Control of Invasive Pests with Entomopathogenic Fungi

Biocontrol of pests is the use of living organisms to reduce pest populations and is an important part of IPM [91]. Entomopathogenic bacteria, fungi, nematodes, and viruses are commonly used microbial control agents, within the framework of biocontrol, for pest management in various cropping systems. While bacteria and viruses are more effective against pests that have chewing mouthparts, entomopathogenic fungi can be effective against a variety of pests. Similar to biocontrol agents, such as parasitoids and predators, entomopathogens can also be released in classical or augmentation approach to control invasive pests [92]. While the objective of classical biological control is permanent establishment of biocontrol agents for self-sustained long-term control of target pests, the augmentation approach represents periodic release of pathogens as biocontrol agents with the expectation that they will multiply and control pests for an extended period, but not permanently. Both the concepts are suitable for microbial control of invasive forest pests by entomopathogenic fungi. Currently, there are over 700 recognized species of entomopathogenic fungi. A majority of economically important species belongs to the order Hypocreales and the new phylum Entomophthoromycota. In general, these fungi are considered excellent candidates for microbial control of many insect pests [93,94]. Several characteristics of entomopathogenic fungi make them an ideal alternative or supplement to chemical insecticide usage. Hypocreales are more general pathogens while Entomopathoromycota are relatively host specific, both with minimal effect on non-target beneficial organisms and are compatible with IPM programs. Entomopathogenic fungi and their metabolites pose no obvious risk to mammalians [95], however tissue infections and allergies can be very rarely observed in immunocompromised individuals [96,97,98]. A further reason in favor of using microbial biocontrol agents is the increasing emergence of resistance in pests to chemical pesticides [99,100]. Among entomopathogenic fungi, hypocrealeans such as *Beauveria* spp., *Isaria fumosorosea*, and *Metarhizium* spp. are available as commercial formulations for inoculative and inundative applications. Entomophthoraleans, such as *Entomophaga maimaiga* Humber, Shimazu, and Soper, are naturally occurring fungi and cause epizootics in pest populations. Microbial control efforts of the invasive forest pests discussed in this article are presented here.

### 3.1. Cypress Aphid Control

**Figure 1:**
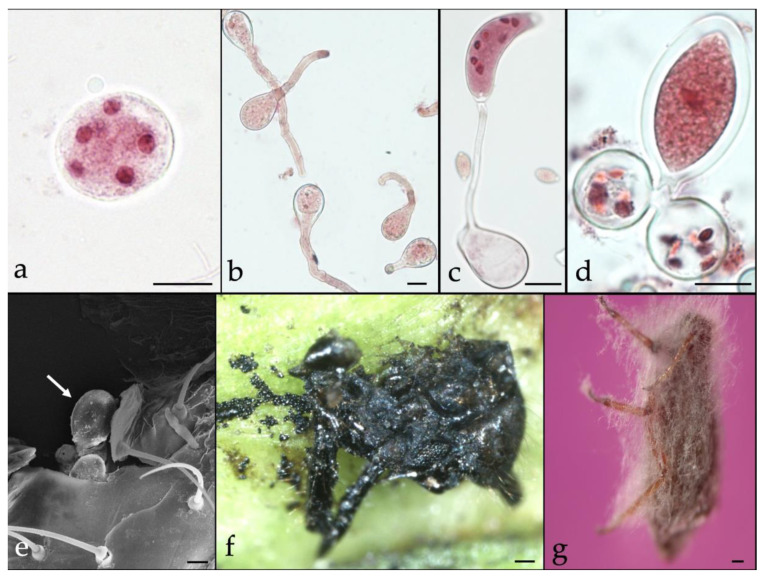
Entomopathogenic fungi, *Neozygites osornensis* (**a**–**f**) and *Lecanicillum attenuatum* (**g**). on *Cinara cupressi*. (**a**). Subspherical hyphal body with visible nuclei (Bar = 10 μm), (**b**). Primary conidia germinating apically (Bar = 10 μm), (**c**). Capilliconidium developed from primary conidium on capillary tube (Bar = 10 μm), (**d**). Zygospore developing by conjugation of two hyphal bodies (Bar = 10 μm), and (**e**). Fully developed zygospore attached on killed aphid (Bar = 10 μm), (**f**). Fungus-killed *C. cupressi* (Bar = 100 μm), and (**g**). Mycelial growth on fungus-killed *C. cupressi* (Bar = 100 μm).

A survey of entomopathogenic fungi of *C. cupressi*, was carried out in southern Chile (project DID 2011-11) between 2007 and 2013. Several strains of *Lecanicillium attenuatum* were isolated and a *Neozygites* species wasreported from naturally infected *C. cupressi* cadavers (Figure 1) in different localities [17,101]. Several laboratory studies around the world demonstrated high levels of mortality in *C. cupressi* with entomopathogenic fungi of the order Hypocreales, especially with *Lecanicillium* sp. [17,102,103,104]. However, field efficacy varies considerably as these fungi are strongly influenced by the environmental conditions such as humidity and temperature. On the other hand, some fungi of the order Neozygitales were cited and collected in different locations in Chile [101,105,106]. For example, *Neozygites turbinata* (Kenneth) Remaudière and Keller and *Neozygites osornensis* Montalva and Barta are highly specific to certain aphids and can cause epizootics in pest populations, and they can also be multiplied as protoplasts or hyphal bodies. However, challenges of in vitro and in vivo production of *Neozygites* spp. limit their use as an augmentation control option [107]. Releasing live aphids infected with *Neozygites* spp. could be an option, as seen with *Neozygites floridana* (Weiser and Muma) Remaudière and S. Keller, for controlling the cassava green mite, *Mononychellus tanajoa* (Bondar), in West Africa [108].

### 3.2. Eucalyptus Weevil Control

**Figure 2:**
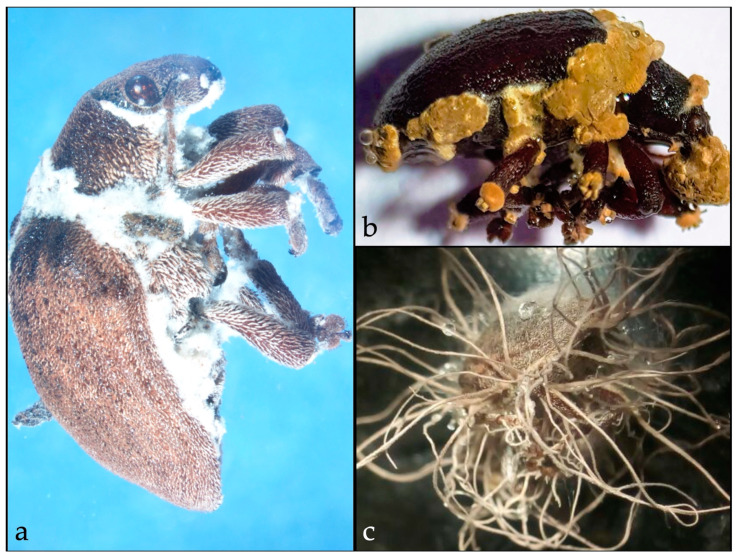
Entomopathogenic fungi on *Gonipterus platensis* infected by. (**a**). *Beauveria* sp. (**b**). *Hirsutella* sp., and (**c**). *Metarhizium* sp.

A survey with the primary objective of discovering entomopathogenic fungi of *G. platensis* was carried out in Chile (project Fondecyt de Iniciación Nº 11160555) between 2016 and 2018. Different species of the genera *Beauveria*, *Hirsutella*, and *Metarhizium* (Figure 2) were found from natural infections in adult *G. platensis* in or when insects were exposed to soil samples containing entomopathogenic fungi (project Fondecyt Regular Nº 1190390). In South Africa, Echeverri and Santolamazza [109] evaluated three formulations of *B. bassiana* and a suspension containing spores of *Metarhizium acridum* against adults *G. scutellatus* under laboratory conditions. *Beauveria bassiana* (strain PPRI 5339) exhibited the highest efficiency, both by contact and ingestion, resulting in 100% adult mortality; thus, appearing to be the most promising strain to promote an IPM program in South Africa.

### 3.3. Gypsy Moth Control

**Figure 3:**
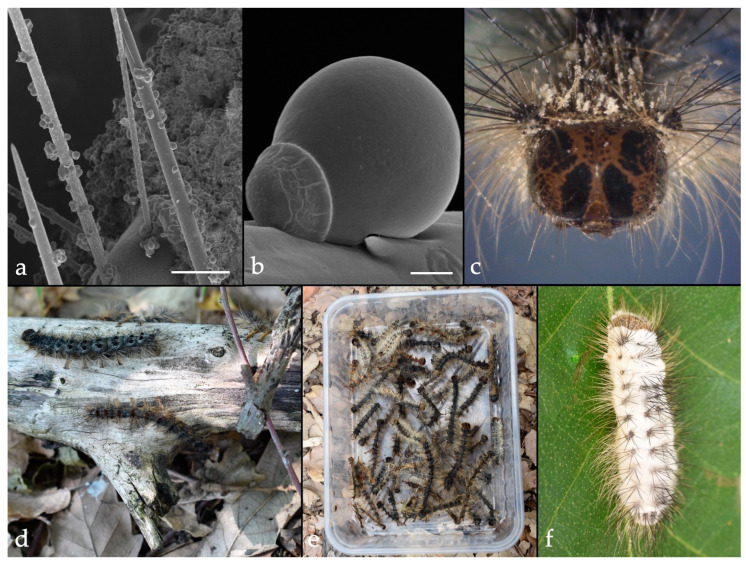
Entomopathogenic fungi, *Entomophaga maimaiga* (**a**–**e**) and *Beauveria bassiana* (**f**) on *Lymantria dispar*. (**a**). Primary conidia attached on setae of killed larva (Bar = 200 μm) (**b**). Primary conidium attached on larval seta (Bar = 5 μm), (**c**). Larval cadaver with emerged conidia, (**d**). Infected-live larvae, (**e**). Fungus-killed larvae collected from a forest, and (**f**). Sporulating cadaver.

*Lymantria dispar* probably has more microbial control options than the other invasive pests reviewed in this article. *Bacillus thuringiensis* Berliner subsp. *kurstaki*, *Lymantria dispar* multicapsid nucleopolyhedrovirus (LdMNPV) and *E. maimaiga* (Entomophthoromycota: Entomophthorales) have been used for controlling *L. dispar* [110,111]. The introduction of *E. maimaiga* from Asia to the United States is a good example of classical microbial control. Following the introduction of this fungus, epizootics by this species predominate in *L. dispar* populations, although low levels of consistent infections by *I. fumosorosea* or occasional infections by *B. bassiana* are also detected [112]. Currently, *E. maimaiga* is the most important host-specific fungal pathogen of *L. dispar* larvae in North America (Figure 3). It was originally described from Japanese gypsy moth *L. dispar japonensis* [113]. In 1910–11, the fungus was intentionally introduced into the United States as a classical microbial control agent, using two cadavers containing resting spores. However, it was not recovered in *L. dispar* populations in subsequent years, and the program was terminated as unsuccessful in 1912 [114]. In 1989, unexpected high mortality of *L. dispar* larvae, caused by *E. maimaiga*, was recorded in the northeastern United States [115]. Molecular studies and models suggest that the *E. maimaiga* strain, now active in the United States, was an accidental introduction after 1971 [116]. At present, the fungus has a major impact on *L. dispar* populations in the United States and reduces defoliation caused by the pest [117,118]. The successful introduction of *E. maimaiga* into North American populations of *L. dispar* inspired its introduction into Bulgaria in 1999 [119]. Surveys conducted in subsequent years confirmed that the pathogen was successfully established and the first epizootics of *E. maimaiga* in *L. dispar* populations were observed in 2005 [120,121,122]. Since 2011, the fungus has been recovered in several countries of central and southeastern Europe [123,124,125,126,127,128,129].

### 3.4. Asian Longhorned Beetle Control

Multiple studies demonstrated the potential of *B. brongniartii*, *M. anisopliae*, and *M. brunneum* Petch in controlling *A. glabripennis* [130,131,132]. Adult longevity and female oviposition of *A. glabripennis* was significantly affected when exposed to non-woven fiber bands impregnated with commercial and native isolates of *B. asiatica* Rehner and Humber and *B. brongniartii* [130]. In a study conducted with *M. brunneum* (strain F52), fungal bands based on agar and two oil formulations and agar-based bands resulted in improved conidial acquisition by beetles and rapid mortality [132]. Similarly, conidial viability and virulence of *M. brunneum* strain F52 was maintained for at least 112 days under field conditions in studies conducted against *A. glabripennis* [133]. Two *M. anisopliae* isolates significantly reduced female longevity and fecundity of *A. glabripennis* and also reduced the eclosion of larvae from eggs deposited by infected females [131]. It appeared that starvation had a similar impact on the survival of *M. brunneum* inoculated beetles compared to imidacloprid exposure [134]. The synergy, however, was not completely due to starvation, as imidacloprid reduced the beetles’ melanotic encapsulation response and capsule area, while starvation did not significantly reduce these immune responses. Their results suggest that multiple interacting mechanisms are involved in the synergy between *M. brunneum* and imidacloprid. Furthermore, it appeared that mature and old females of *A. glabripennis* were more susceptible to *M. brunneum* than males of equal ages, and more females had detectable fungal blastospores in their hemolymph compared to mature and old males [135]. Also, laboratory conditions demonstrated that *M. brunneum*-infected *A. glabripennis* does not exhibit behavioral fever (elevating body temperature by exposing to a heat source to ward off fungal infections [136]. Bioassays conducted in the United States showed that the Japanese commercial strain of *B. asiatica* and the commercial strain F52 of *M. brunneum* were more virulent than two North American *B. brongniartii* isolates against *A. glabripennis* [137]. In Japan, basic experiments were performed in order to develop biorational control of cerambycid beetle, including *A. malasaica* (=*chinensis*). Local strains of *B. brongniartii* incorporated in non-woven pulp fabric sheets, or polyurethane sheets, were applied around branches and tree trunks against cerambycid adults emerging from trees [138,139]. Between 46% and 100% adult mortality was observed when adults were delivered on polyurethane sheets wrapped around the lower portion of the trunk [138].

### 3.5. Emerald Ash Borer Control

**Figure 4:**
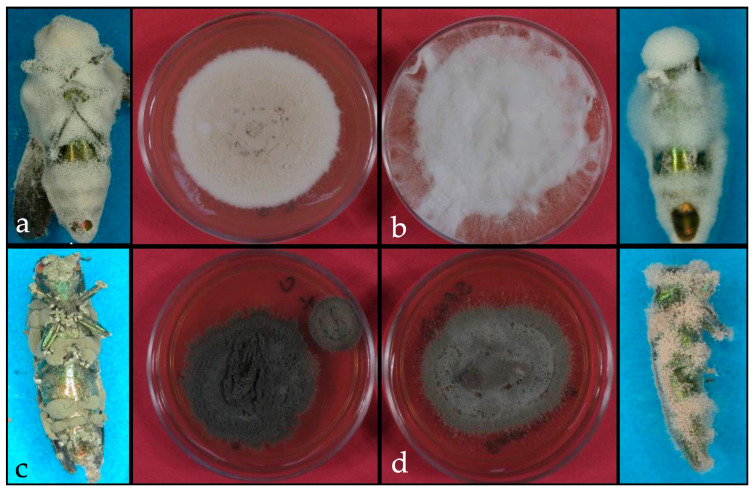
Entomopathogenic fungi on *Agrilus planipennis*. (**a**). Infected adult and a culture of *Beauveria* sp., (**b**). Infected adult and a culture of *Lecanicillium* sp., (**c**). Infected adult and a culture of *Metarhizium* sp., and (**d**). Infected adult and culture of *Paecilomyces* sp.

**Figure 5:**
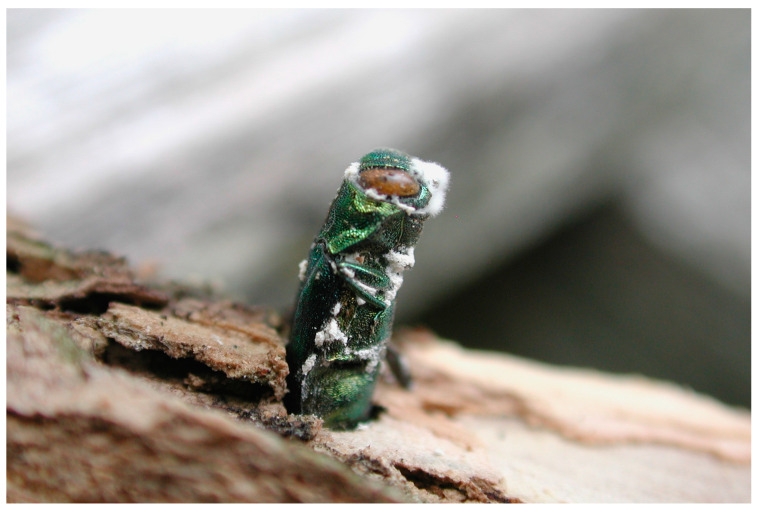
Adult of *Agrilus planipennis* infected with *Beauveria bassiana* as a result of pre-emergence field cage trials.

*Agrilus planipennis* is attacked by several species of entomopathogenic fungi. However, *B. bassiana* appeared to be a potential microbial control agent based on multiple studies. Spray applications of the commercial formulation of *B. bassiana* strain GHA to the trunks of ash trees, especially before the adult emergence in summer, reduced adult longevity and female fecundity and delayed larval development (Figure 4) [140]. Castillo et al. [141] also found that conidial sprays of *B. bassiana*, on the ash bark before adult emergence, remain viable enough to be a significant mortality factor. A study conducted in Canada demonstrated the potential of using insect traps equipped with *B. bassiana* conidia as an attract-and-kill strategy for *A. planipennis* [142]. Two additional genera demonstrated pathogenicity in laboratory conditions. *Isaria farinosa* (Holmsk.) Fr. and *Purpureocillium lilacinum* (Thom) Luangsa-ard, Houbraken, Hywel-Jones and Samson infected adults with high mortality rates (75% and 51%, respectively) under laboratory conditions [143] (Figure 5). Lyons et al. [144] evaluated the use of fluorescent dyes as a cost-effective method of tracking dispersal by *A. planipennis* of *B. bassiana* isolates that were introduced by using an autocontamination device. Neither of the two fluorescent dyes tested (Arc Yellow and Aurora Pink, DayGlow Color Corp) interfered with fungal germination or growth, nor did they affect survival of beetles in the laboratory or affect virulence of the fungus in bioassays. The dyes persisted outdoor exposure for at least 10 days on dead beetles in sticky band traps, and for at least 14 days on pouches inside autocontamination traps. Duplicate field trials in late June–early July 2012, using autocontamination traps containing powder-dusted fungal pouches in ash-borer infested plantations in southwestern Ontario showed fluorescent dyes in 8.0% of the 4010 beetles captured in nearby green prism and sticky-band traps. However, only half (46.2%–57.8%) of the beetles with dyes carried viable fungal conidia, as determined by plating of beetle rinses, possibly as a result of patchy growth of fungal isolates and reduced conidia production on pouch surfaces during the 16-day trapping experiment.

### 3.6. Oak Lace Bug Control

Effective control methods for *C. arcuata* are limited and include mostly application of oils or contact insecticides on infested ornamental oaks [145]. To date, no biocontrol programs were used to regulate *C. arcuata* infestations. However, a pilot study has been initiated in Turkey recently to test ten entomopathogenic fungi against both nymphs and adults of *C. arcuata* under laboratory conditions [146]. Entomopathogenic fungi of the genera *Metarhizium*, *Beauveria*, *Isaria*, and *Myriodontium* were included in this study. All fungal strains were able to infect the pest after application of 1 × 10^7^ mL^−1^ conidial concentration, but *B. bassiana* strain was very pathogenic to both nymphs and adults, with 80% and 90% mortality within 14 days of exposure, respectively.

## 4. Concluding Remarks

Although a variety of IPM tactics are used for controlling forest pests, chemical pesticide use is still one of the primary choices around the world. Considering the environmental impacts and the risk of resistance, alternative control options are always important. While application of biopesticide formulations can be expensive, they play a critical role in pest suppression, especially in areas close to urban dwellings, waterbodies, and other such sensitive locations. Natural epizootics by fungi and viruses can help with forest pest control a great deal and understanding the disease dynamics will be useful in delaying pesticide applications or developing integrated strategies. Since several forest pests are invasive and continue to spread in their new homes, or to other areas, the knowledge of microbial control potential with entomopathogenic fungi will contribute to their sustainable management.
